# Healthcare Professionals' Experiences with Functional Independence Measure (FIM) as a Structured Framework for Interprofessional Team Meetings in Danish Stroke Rehabilitation: A Qualitative Cross-Sectoral Collaborative Study

**DOI:** 10.1155/2023/6660296

**Published:** 2023-09-27

**Authors:** Jon Damsager Lauesen, Kristian Larsen, Johanne Laursen Lykke, Mona Christensen, Christian Hedelund Arens, Hanne Bigum

**Affiliations:** ^1^Neurorehabilitation Copenhagen, Copenhagen, Denmark; ^2^Department of Public Health, University of Copenhagen, Denmark; ^3^OsloMet - Oslo Metropolitan University, Oslo, Norway; ^4^University Hospitals Centre for Health Research (UCSF), Denmark; ^5^Department of Neurology, Copenhagen University Hospital, Bispebjerg and Frederiksberg, Copenhagen, Denmark; ^6^Department of Physio- and Occupational Therapy, Copenhagen University Hospital, Bispebjerg and Frederiksberg, Copenhagen, Denmark

## Abstract

**Purpose:**

An ethnographic and phenomenological mapping of the experiences of healthcare professionals with the functional independence measure (FIM) in stroke rehabilitation.

**Methods:**

This is a cross-sectoral qualitative study with triangulation of data from two focus group interviews, 15 individual interviews, and 11 participant observations of FIM assessments performed by six different healthcare professions in interprofessional teams. FIM assessments were performed at hospital and in a community rehabilitation centre as interprofessional meetings with a local facilitator certified in FIM.

**Results:**

Three overarching themes, learning space, improved interprofessional collaboration, and transferability, emerged from the data. The use of FIM within the provided structures established an environment that allowed the various healthcare professionals (HCP) to learn with, about, and from each other. This is perceived as promoting interprofessional collaboration and enhancing patient-specific knowledge within the interprofessional team. The established patient-specific knowledge is specific to the individual team and is difficult to transfer intraorganisationally and across sectors.

**Conclusion:**

FIM was a catalyst for improved interprofessional knowledge transfer and interprofessional collaboration within the individual teams, but intraorganisational and cross-sectoral dissemination of patient-specific knowledge was limited.

## 1. Introduction

Stroke causes impairments within consciousness, motor, sensory, and cognitive functions [1, 2]. The complexity of impairments following stroke renders rehabilitation a multidimensional task, where admission to specialised units is associated with favourable patient outcomes [3–5]. This multidimensionality places emphasis on interdisciplinary collaboration.

In the Danish healthcare system, patient pathways are divided into different phases, with hospitals treating acute illness and initializing rehabilitation followed by the local municipalities administering further rehabilitation at rehabilitation centres and outpatient clinics. Based on Danish national patient registers, 12,000-13,000 annually are admitted to hospital due to a new stroke with a decrease in length of stay at hospital within the last decade [6]. As a result, early rehabilitation is increasingly provided by the community-based rehabilitation centres. In the last thirty years, there has been a large increase in the number of specialisations within healthcare professionals, furthermore in treatment options [7]. This has led to greater complexity and a simultaneous increase in procedural standardisation [8], with additional documentation requirements and new forms of management permeating the field [9, 10]. The added complexity generates the need for interdisciplinary coordination and collaboration between healthcare professions and between sectors, but at the same time, the professions work increasingly separately, divided, and specialised [11]. Consequently, with the growth in scholarly attention paid to how healthcare professionals (HCP) collaborate, terms such as multiprofessional, transdisciplinary, and interprofessional have been adopted from the social sciences and used in the healthcare literature to describe various forms of collaboration [12, 13]. The collective use of outcome measures across sectors is believed to facilitate knowledge transfer and bridge gaps [14]. Various specific and general instruments and outcome measures have been developed to address the multitude of impairments that can occur after stroke [15, 16]. FIM is a valid and reliable [17–20] multidimensional outcome measure [21] used by interprofessional teams to assess patients' independence or need for assistance. It is used in settings from acute to community rehabilitation and across various patient groups with neurologic disorders and other diseases [19, 20, 22]. It assesses 18 motor and 5 cognitive functions which makes it particularly relevant in stroke rehabilitation. The multidimensionality of the FIM calls for interprofessional usage and therefore facilitates interprofessionality. However, little is known about how interprofessional teams, in stroke rehabilitation, perceive FIM influencing their interprofessional collaboration.

The aim of this study is to explore experiences among HCPs regarding how FIM affects interprofessional collaboration and cross-sectoral knowledge transfer. We examine whether FIM constitutes a structured framework influencing interprofessional and cross-sectoral collaboration. To the best of our knowledge, this has not been explored in prior studies.

## 2. Methods

### 2.1. Study Design

This qualitative study is part of a mixed methods study examining the application of FIM with the aim of strengthening interprofessional and cross-sectoral collaboration in stroke rehabilitation. In the context of this study, cross-sectoral refers to hospital and municipality. The study protocol was published at clinicaltrials.gov (NCT04564820) prior to participant enrolment.

In this study, we applied an ethnographically and phenomenologically descriptive and explorative approach to elucidate the experiences of HCPs with FIM in stroke rehabilitation. We applied Bourdieu and Wacquant's [23] reflections on double objectivity to underpin the epistemological principles. In those reflections, objectivity of the first order is empirical data (ethnography—observation) of bodies in materiality, and objectivity of the second order (phenomenology—interviews) focuses on the experiences of the subjects. To gain a more differentiated and comprehensive understanding, we used triangulation in the data collection [24] by incorporating focus group interviews, subsequent individual interviews, and observations of HCPs during facilitated FIM assessments. We utilised multilevel triangulation among the three primary researchers to ensure differences in profession, organisation, knowledge, and clinical practice within the field of neurorehabilitation.

### 2.2. Setting

D'Amour and Oandasan define interprofessionality as “(…) the development of a cohesive practice between professionals from different disciplines. It is the process by which professionals reflect on and develop ways of practicing that provides an integrated and cohesive answer to the needs of the client/family/population” [25]. We apply this as the underlying basis for our application of the term interprofessional collaboration within the World Health Organization's Framework for Action on Interprofessional Education and Collaborative Practice [26], the gold standard for collaborative work among HCPs from different professions. In this framework, health workers from different professions learn about, from, and with each other to enable effective collaboration and improve health outcomes. The study is a cross-sectoral collaboration between a hospital and a local community neurorehabilitation centre (CNC). Patient-specific interprofessional teams of HCPs at both locations conducted separate FIM assessments of patients whose rehabilitation was initiated at the hospital before being continued at the CNC. In this study, FIM assessments refer to interprofessional team meetings where the HCPs congregate and collaborate on assessing all items of the FIM based on relevant observations from daily rehabilitation. Assessments were performed within 72 hours from admittance and at discharge from both the hospital and the CNC. Due to managerial decisions, FIM was administered differently at the hospital compared to the CNC. At the hospital, the interprofessional team participating in the assessments comprised HCPs introduced to FIM and who also provided daily rehabilitation to the patient being assessed. At the CNC, the interprofessional team consisted of HCPs trained in using FIM, where only some of them also provided daily rehabilitation for the patient being assessed. To assist the interprofessional teams and ensure validity, assessments were facilitated by one or two clinical experts in neurorehabilitation certified in the use of the FIM. The facilitator did not necessarily possess specific knowledge of the patient being assessed but administered the scoring based on the information that the interprofessional teams provided. The facilitators' responsibility was further to engage the HCP's in the assessment and ensure active participation from each profession while establishing an interprofessional plan for the patient.

**Figure 1:**
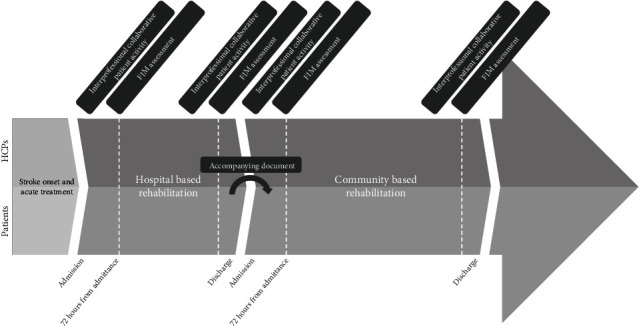
Patient pathway and timing of FIM assessment-related activities for HCPs. HCPs: healthcare professionals; FIM: functional independence measure.

One hour was scheduled for the assessments, which took place outside the ward to avoid any disruptions due to other patient-related tasks. At least one day prior to patients being assessed, participating HCPs, their coworkers, and managers were told when and where the assessments would take place. To ensure that the various professional groups had essential FIM-related observations, two HCPs from two different professions performed at least one interprofessional collaborative patient activity, e.g., assisting with showering, getting dressed, or other FIM-related activities, prior to assessments (Figure 1).

After FIM assessments, points of attention, suggestions for actions, and concerns from the interprofessional team were documented in the electronic patient records. Prior to discharge, the HCPs provided an accompanying document that followed patients from the hospital to the CNC to supplement the numerical FIM scores with nuanced information.

HCPs were introduced to the use of spiderweb charts as a visual aid to help explain FIM assessments to patients and their relatives. These charts showed current and former FIM scores from each item, thereby illustrating items with progression and items which could be targeted for future goals.

### 2.3. Participants

HCPs at the hospital and CNC were recruited, from September to November 2021 from among staff performing FIM assessments. We applied purposeful sampling [27] to ensure maximum distribution between healthcare professions at both sites. Two nurses, four nursing assistants, two speech and language therapists, two occupational therapists, one neuropsychologist, and four physiotherapists participated in the focus group and individual interviews. Of those, 12 were female and three male, with ages from 25 to 58 years (median: 34) and with <1 to 23 years (median: 5) of experience within neurorehabilitation. Participants received information on the study and provided written consent to participate.

### 2.4. Empirical Data

The research team developed an interview guide containing open and exploratory questions based on their collective specialist knowledge on stroke rehabilitation, FIM, and qualitative research interviews. Semistructured focus group interviews were conducted to shed light on interprofessional positions and structures since they make it possible to challenge and support the opinions being expressed and to expand the interview to areas the interview guide did not cover [28]. Subsequently, a few additional questions were added to the interview guide in advance of the individual interviews. Semistructured individual interviews were conducted to ensure that respondents were freely able to express and elaborate on their own opinions without being silenced by group norms [29]. Field notes were taken during interviews and used during data processing to assist interpretation of the data material. Three different people conducted the interviews, one of them an experienced interviewer. To mitigate deficiencies in the data collection due to the level of experience and to allow familiarisation with the interview guide, two interviewers took part in both focus group interviews, one of whom was the experienced interviewer. In the initial individual interviews performed by less experienced interviewers, the one with the least experience was present as an observer. Participant observations of HCPs during FIM assessments were conducted to identify social phenomena and to contrast and support experiences expressed during both types of interviews [30, 31].

The triangulation of data between the two different types of interviews and observations provides an opportunity to examine how participants construct various levels of meaning in their work and how these levels work together or become coordinated [24]. Two semistructured focus group interviews, fifteen subsequent semistructured individual interviews, and 11 participant observations were conducted (Table 1).

The conducted participant observations of FIM assessments were attended by two to eight HCPs with a total of 42 HCPs across all observations. As some HCPs were present at multiple observed FIM assessments, a total of 32 individual HCPs were observed (four nurses, five nursing assistants, three speech and language therapists, nine occupational therapists, two neuropsychologists, eight physiotherapists, and one neuropedagogue).

### 2.5. Validation

Digital audio recordings from the interviews were transcribed verbatim. Participant observations were video recorded, with data subsequently added to a predefined observation chart. Two from the research group transcribed the focus group interviews, while research assistants transcribed the individual interviews and reviewed the video recordings. To ensure transparency, three researchers (JDL, KL, and HB) listened to every interview and reviewed all observations to ensure that at least two researchers had reviewed all the data. Transcriptions were read, organised, coded, and thematised inductively using Braun and Clarke's six-step approach [32] in a recursive process. During the analysis, the researchers met frequently to discuss the coding and review suggested themes until complete agreement was obtained (Table 2). Themes were further reviewed for independence or connection and organised in overarching themes.

To ensure content stability and linguistic agreement during translation, selected quotes were translated from Danish to English by MC, a physiotherapist and native speaker of Danish with 15 years' experience in neurorehabilitation, with six of those working in England in the National Health Service.

### 2.6. Ethical Considerations

The study fulfils the principles of the Helsinki Declaration [33]. The Danish Data Protection Agency was informed (ID: P-2020-1031), and the study was presented to the Capital Region of Denmark's local ethics committee (ID: H-20036071) who determined that their approval was unnecessary as no patient directed interventions were involved.

## 3. Findings

The empirical data support the three interconnected, overarching themes: learning space, improved interprofessional collaboration, and transferability. FIM assessments support the various professions in hearing and learning one another's professional concepts and analyses in relation to the patient. It thereby establishes a learning space that improves interprofessional collaboration. The multiple perspectives and experiences concerning patient phenomena are not just confined to a single interprofessional team but disseminated horizontally and across sectoral boundaries.

### 3.1. Overarching Theme I: Learning Space

The learning space, shaped by individual and organisational structures, was explored and described based on five subthemes: time, facilitator, knowledge, agenda, and language. These subthemes, which are described in more detail below, emerge in our analysis as constituent parts that are mutually dependent and interconnected.

#### 3.1.1. Time

The subtheme *time* emerges as somewhat of a paradox. The time allocated for conducting the FIM assessments provides access to the collective knowledge of the interprofessional team, allowing the assessment to go beyond simply being a quantification of functional independence. The teams have the time to immerse themselves in details of the various observations and emerge with a higher understanding of the patient's resources and difficulties.

*(…) I think that the biggest advantage is that we actually have a whole hour to, you know, delve into the patient, so to speak, in an interprofessional setting. You know, where you can really, where you are able to share thoughts and details about the patient. (…)* (Occupational therapist 1, individual interview)

A clear consensus emerges that the time allocated for the assessments is instrumental to the perceived value of the FIM. The HCPs found that the time spent was worthwhile, though not without consequences since it simultaneously meant spending valuable time away from their other patients and duties. They debated whether the time spent constituted extra time or was made up for due to more effective horizontal coordination.

*I think that the discussion about time is tricky because we talk about the patients all the time, in between (…) sometimes I spend time with a nurse first, and then the physio. And then we need to get a hold of the neuropsychologist, and suddenly I've spent 15 minutes here, 15 minutes there, and another 15 there, with this [FIM] we are all here, (…) so I think time is a tricky aspect (…). But I think it's a good use of time in relation to interprofessional dialogue.* (Occupational therapist 1, focus group interview 1)

Even though many participants mentioned this dichotomy surrounding the consumption of time, there was a general desire to expand FIM to a wider range of patients than those included in the study. HCPs perceived that this would require a balancing act between value, priorities, and what is possible in practice.

#### 3.1.2. Facilitator

*Facilitator* is an important subtheme because the facilitator influences the mood and tone of the learning space by establishing tangible and intangible rules of conduct to ensure a welcoming and safe environment in which each profession is a valued asset. Their approach to managing the space safeguarded that all manner of issues was discussed, which had a significant impact on how HCPs contributed and spoke with one another.

*When the person [facilitator] steps inside the room and we [participants] feel here's someone we can lean on (…) That's actually what makes it easier for us to contribute. That's very evident.* (Neuropsychologist, individual interview)

The participants experience that the facilitator supports and challenges the current practice during the assessment by asking questions and encouraging reflection, thereby facilitating the HCPs in incorporating their monoprofessional knowledge into the interprofessional collaboration while shifting the focus from profession to patient.

#### 3.1.3. Knowledge

The subtheme *knowledge* pertains to the level of patient-specific knowledge HCPs possess when performing the FIM assessment. The HCPs express that the timing of the assessment and prior interactions with the patient influence their level of knowledge. The timing is associated with two distinct experiences. First, participants at both the hospital and CNC felt that they lacked sufficiently detailed knowledge about the patient during initial assessments. There is definitive consensus among them that the timeframe of doing the first assessment within 72 hours of admission, as stipulated in the FIM manual, was too short. This might be because of organisational challenges from shifts that challenge the continuity of the personal. This brief window of 72 hours left them feeling uneasy and doubting whether they were doing the patient justice in their assessment, which was accompanied by concerns about the actual validity of the assessment.

*But I think it's really, really early (…). The patient gets a lower score because you do not know the patient, or you have not seen the patient brush their teeth. Or you have not seen the patient shower or something else. I think the initial assessments are a bit of a mixed bag.* (Nurse 1, individual interview)

Second, at the hospital, HCPs had a mixed reaction regarding the FIM assessment at discharge. On a positive note, they talked about the benefits of having in-depth knowledge about not only the patient but also the *person*, which they applied during the assessment. Moreover, it drove their interprofessional planning and goal setting in terms of pushing the potential for a more coordinated and focused rehabilitation. The downside was that the planning felt pointless because the patients were about to be discharged.

*(…) once we have done the final FIM assessment and made some really good observations and agreed on some things, then they get discharged the next day. That's a bit of shame. It would've been cool if we could have done it two weeks before and then applied that knowledge.* (Physiotherapist 1, focus group interview 1)

Perceptions concerning the utility of interprofessional collaborative patient activities differed depending on the individual HCP's prior interactions and, thus, knowledge of the patient. When HCPs possessed knowledge relevant to performing the FIM assessment, the interprofessional collaborative patient activities were perceived as superfluous. Contrary, when obtaining knowledge was required, the interprofessional collaborative patient activities were perceived as meaningful. In these cases, they enabled a better platform for interprofessional dialogue and situational knowledge transfer between professions.

#### 3.1.4. Agenda

The *agenda* subtheme was identified based on participant observations. They showed that FIM provided a framework for interprofessional discussions on the patient's current functional abilities. It organised the dissemination of knowledge within the interprofessional team by ensuring a stepwise focus on each item and thus a framework for sharing observations.

*(…). Uh, so yeah, I think so. It can provide structure and actually remind you that, hey, there's also this aspect too. (…)* (Speech and language therapist 2, individual interview)

The HCPs said that FIM ensured a systematic approach, the meticulous assessment drawing attention to areas that might otherwise have been overlooked. The structure provided clarity and assisted the HCPs in prioritising and deciding what they should focus on together, which promoted team synchronisation that transferred into collaborative goal setting.

#### 3.1.5. Language

The last subtheme, *language*, was chosen because individual professions and the field of rehabilitation are each associated with distinct and specific terminology and diction. Throughout our observations and interviews, the participants notice a change in their language as it became more synchronised. The learning space facilitated linguistic alignment across professions but also a joint, conceptual understanding of specific words.

*For example, if you say: “…independent in the bathroom”. Then all of the professions know what we are talking about, then there's a mutual understanding of what that means.* (Physiotherapist 1, focus group interview 1)

They generally find that they have been given a common language that broadens the conversation and enhances understanding during interprofessional discussions, with the added benefit of increasing trust between professions and promoting cohesion.

Through these five subthemes, the “learning space” is formed. The facilitator sets the frame through the prescribed agenda and allocated time, followed by the appreciative tone. Within that frame, the HCPs engage in shared clinical reasoning, slowly shaping their common language and knowledge.

### 3.2. Overarching Theme II: Improved Interprofessional Collaboration

The second overarching theme, improved interprofessional collaboration, is evident throughout the empirical data, though no prominent subthemes emerged. Improved interprofessional collaboration is what the HCPs themselves perceive as the true value stemming directly from the established learning space. They use new strategies, addressing problems collaboratively and solving them differently than before.

*(…) I think there's something particularly exciting about this enthusiasm, which can also appear during assessments with this group dynamic which lets everybody leave with a feeling of euphoria because we solved difficult everyday life issues together.* (Neuropsychologist, individual interview)

This change affected the assessments and extended into daily tasks, where the participants found that reaching out and asking for help across professions was easier. The empirical data on the direction of who learns from whom indicates the continued presence of hierarchical structures.

*But if I see a minor challenge that I do not know how to handle I reach out to a physio or an OT and tell them: “You really have to show me. Please help me so I can do the same”.* (Nurse assistant 2, individual interview)

Mainly nurses and nursing assistants express that they obtain generalisable knowledge from the learning space that changes the way they perform or view their approach during rehabilitation. They recognise how their monoprofessionality applies to rehabilitation and is part of the interprofessional collaboration, which is associated with enhanced motivation and job satisfaction.

*But it's very clear, when we are doing the FIM assessment, that I play a role because there are many of the things concerning bladder control, bowel control, and initiative across the course of the day outside of exercise, where I'm the one who knows about them.* (Nurse assistant 1, individual interview)

Where nurses and nursing assistants mainly describe improvements in their skills, other professions instead emphasise improved interprofessional collaboration and taking a more unified approach. We found that the physio- and occupational therapists, neuropsychologists, and speech and language therapists felt that they hear and listen to the nursing staff differently in connection with the FIM assessment, where their contributions sought-after and appreciated.

*I think that they have incredibly much to offer but you do not always get to hear it. I talk more with the physio-OT group. (…) I think it's nice that the nurses become more involved. I missed what they could bring to the table before.* (Speech and language therapist 1, individual interview)

Our results show that the interprofessional teams use the time and collective knowledge to decipher not only the patients' difficulties but also to discuss why and how to address them. They determine potential focal points for future rehabilitative efforts and transfer them from the FIM assessments to meetings where the patient and relatives participate and are involved in goal setting.

*But I think that because we know these things from the FIM assessment, where the patient has problems, and how we can help the patient with those problems. When we know these two things it's a lot easier to assist the patient in setting realistic goals (…). And that's exactly what we have been missing (…).* (Physiotherapist 1, individual interview)

We find that the enhanced understanding and knowledge of the different professional skillsets and positions promotes interprofessional cohesion and brings the team closer together. They collaborate on determining goals and strive for a unified approach to everyday tasks, their joint efforts further heightening their perception of improved interprofessional collaboration.

### 3.3. Overarching Theme III: Transferability

The third overarching theme relates to the HCPs' experiences pertaining to the dissemination of patient-specific knowledge from the learning space to other settings, professionals, and across healthcare sectors. Two subthemes emerged as experiences with transferability converge around either intraorganisational or cross-sectoral knowledge transfer.

#### 3.3.1. Intraorganisational Knowledge Transfer

For this subtheme, the empirical data shows that some patient-specific knowledge is transferred from the interprofessional team to other HCPs within the organisation as well as to patients and their relatives. However, this transfer is perceived as difficult and limited.

When explaining FIM assessments to patients and relatives, the HCPs stated barriers such as time, priorities, and applicability, against using the created spiderweb charts as graphical aids. When used in selected relevant cases, the HCPs experienced the spiderweb chart or transformation of the data into columns as useful and well received.

*I brought it [spiderweb chart] to PPR [participatory patient rounds], where the [patient's] daughter was very interested in it and where I kind of took it step by step all the way around the spiderweb and explained, well, this is this and that is that. (...)* (Physiotherapist 1, individual interview)

We found that profession-specific organisational demands related to how the various professions are deployed and used influence dissemination of knowledge differently depending on the specific profession and sector. Especially at the hospital, nurses and nursing assistants' stability in the interprofessional team is challenged by working in shifts and the allocation of competencies depending on the patients' current condition.

*What's more you have to care for a new patient where an [FIM] assessment might have been completed, where you have to absorb that knowledge and then a new patient the next day, so it's also a crazy, so to speak, a crazy demand on the nurses placed in that situation to be flexible and adapt.* (Neuropsychologist 1, focus group interview 1)

The HCPs express that transfer of knowledge to other professionals outside the interprofessional team occurs but is not without challenges.

#### 3.3.2. Cross-Sectoral Knowledge Transfer

The other subtheme in this section is cross-sectoral knowledge transfer, which in this study is characterised by serial dependency in which one sector is the conveyor and the other the recipient of information. In this context, the mutual use of an outcome measure is perceived by the recipients as bridging the gap between sectors by creating a common ground and aspects on which to communicate.

*(…) I know that we assess on the same level. I mean with the same outcome measure, right. So, this creates a mutual, in some way, you know a better sharing of information or something. I definitely feel that because it provides a mutual language in some ways. (...)* (Physiotherapist 3, individual interview)

The empirical data, however, show a reciprocal relation between data transferability and data value. Information on the numerical FIM scores is easily transferred from one sector to the other but is represented in the data as of lesser value to the HCPs than the associated knowledge behind the score.

*Well, the number alone hardly tells you anything; it's definitely the comments [accompanying document] that are crucial, along with the conversations we have, that pay off.* (Physiotherapist 1, focus group interview 1)

When the patients were transferred from the hospital to the CNC, the numerical scores were supported by an accompanying document containing explanatory narrative. Although participant observations confirm that the accompanying document was presented to the respective interprofessional teams, recollection of these during interviews was vague and rarely seen as carrying much weight. This inability to clearly remember the document represents a missed attempt on transferring knowledge from the learning space in one sector to the other.

## 4. Discussion

In this study, we used FIM as an outcome measure but investigated how this would affect interprofessional and cross-sectoral collaboration. We mapped the experiences of HCPs and found that they perceived FIM assessments as facilitating improvements in interprofessional collaboration. In the following, we will discuss our findings as they pertain to learning through practice, profession, and disciplinarity, as well as policy and organisation.

### 4.1. Learning Space

FIM assessments were added to everyday tasks and generated the allocation of considerable resources in terms of staff and time. With an increased demand for productivity, these extra expenditures should be counterbalanced or outweighed by other factors. The FIM assessments in our study could be viewed as a type of interprofessional meeting where the formal use of FIM as an outcome measure provided an agenda for structured discussions and documentation that necessitated premeeting preparation and were chaired by a facilitator, features all of which Tyson et al. [34] described as associated with effective team meetings. The communication during FIM assessments may have decreased the need for everyday ad hoc communication and improved planning and goal setting, making up for the time spent on it. Further, with FIM, an educational, material, and intellectual space was suddenly created by organisational structures such as time, setting, and the demand for interprofessional attendance. These structures along with a set agenda and a trained facilitator strongly aided the HCPs in engaging in interprofessional collaboration that facilitated the establishment of a common language and horizontal coordination. From the literature, we know that the interrelatedness of time, sociomateriality, and common professional activities encourage learning among healthcare professions [35]. In our study, this familiarisation and relational interprofessional socialisation promoted additional respect, reduced hierarchical thinking, and enabled learning with, about, and from each other, which is at the core of WHO's theoretical framework for Interprofessional Education and Collaborative Practice [26]. Our study contributes by showing that the collective knowledge acquired was a major driver of the enhanced interprofessional collaboration, the outcome that the HCPs perceived as the greatest benefit. This collective knowledge acquired encompasses the type of shared conceptualisations and development of a common language that Greenway et al. [36] determined may enhance understanding and practice in stroke rehabilitation. Although the use of FIM can be confined to simply a numerical outcome measure, we argue that with the proper organisational structures, FIM assessments may represent a practice-based supplement to formalised educational programs on interprofessional collaboration and practice.

### 4.2. Improved Interprofessional Collaboration

With the establishment of stroke units, the benefits of collaboration between the various professions have become increasingly apparent. At the same time, the general transformation of the healthcare field [7, 8] has affected the healthcare professions, making them subject to increased specialisation, separate work processes, efficiency requirements, and an emphasis on the use of monoprofessional outcome measures [37]. These institutional structures limit space for collaboration and increase demand for productivity, which according to Abbott [38] makes professions more isolated since they find it hard to deviate from their monoprofessional approach because they must maintain productivity. This can be understood through the process of socialisation of professions presented by Clark [39], where organisational and educational development of a strong monoprofessional identity can become a barrier for interprofessional collaboration [39, 40]. In the struggle to maintain their autonomy and identity, professions also maintain a profession-specific framework and culture resulting in healthcare services that are still, at times, siloed and hierarchically divided [41]. It minimises the possibilities for the HCPs to prioritise teamwork, thus making interdisciplinarity a desired standard rather than the actual practice [11, 42]. Our findings show that structured FIM assessments as conducted in this study help decrease siloes because the learning space provided a better understanding of the various profession contributions and created cohesion within the interprofessional teams.

To improve collaboration between different healthcare professions, Danish HCPs working with neurorehabilitation have adopted WHO's framework for Interprofessional Education and Collaborative Practice [26] over the last decade. In this study, we did not dispute the HCPs' perception or use of the term interprofessional collaboration nor whether they were correctly aligned with its definition. As the healthcare field is in transition [43, 44] the way healthcare is applied changes and moves back and forth across a continuum towards interdisciplinarity [45]. Our findings show that FIM promotes interprofessional collaboration, indicating that it advances teamwork further along the continuum towards the perceived gold standard of true interprofessional collaboration.

### 4.3. Transferability

Organisational, legislative, and political demands on the healthcare system create a system that forces HCPs to repeat screenings and tests as the patient transfers between hospitals and sectors [9, 10]. The ramifications of this were evident in our study, where two FIM assessments were performed within the short time span of patients being discharged from the hospital and admitted at the CNC. As we found that the HCPs at the hospital said they felt this was redundant, while HCPs at the CNC expressed a lack of sufficiently detailed knowledge, it mandates consideration whether assessments at both ends are clinically relevant or cost-effective. Furthermore, numerical FIM scores are easily sent from one sector to the other, which means they do not represent a logistical hinderance.

Information such as test results can be effortlessly transferred, but the dissemination of related knowledge pertaining to what these results mean or how they should be interpreted is another matter. Studies on the transfer and exchange of knowledge between researchers and policymakers show that knowledge transfer comprises more than simply passing on information [46]. We found that the cross-sectoral transition caused a complete deconstruction of the established learning space due to the change of location and personnel. This deconstruction and subsequent need to establish a new learning space entailed a discontinuity in the knowledge and a barrier to cross-sectoral knowledge transfer that was difficult to alleviate. In this study, in an attempt to navigate this gap, we provided an accompanying document that elaborated on the FIM score. However, similar to other studies, the written information primarily resulted in information transfer and not knowledge transfer [47, 48]. A key reason for this may be that actual knowledge transfer requires interpersonal interaction over a sustained period [46].

### 4.4. Methodological Considerations and Limitations

In our study, a researcher with many years of experience and year-long socialisation into the language, culture, and organisation of stroke rehabilitation conducted most of the interviews. This insider perspective was counterbalanced by the position of the other researchers, enhancing the overall trustworthiness of our findings [49]. In our study, the composition of the research team and applied methodology provided the benefit of detailed field knowledge while mitigating potential biases encountered in other ethnographical studies, where researchers are positioned in the field of research [50].

The combination of two types of interviews, participant observations, observation data sheets, and field notes was essential to our phenomenological and ethnographic approach. The triangulation of methods and researchers provided a unique look into the complexity of the meaning generated intersubjectively. Without the participant observations on the FIM assessments, we would have been left with the understanding that only data on the FIM scores, and not the accompanying document with supplementary information, had been transferred from one sector to the other. The use of participant observations changed our knowledge of what was conveyed and hence altered our interpretation. Notably, during the research process, we used inductive and exploratory ways of constructing empirical data but simultaneously also employed more deductive and theoretically inspired approaches, which is in line with abductive analysis [51].

No pilot testing of the interview guide was conducted before application in the focus group interviews. The focus groups did not present aspects that were not covered in the interview guide, and therefore, the same interview guide was applied for the individual interviews.

The purposeful sampling was applied to allow possible profession-specific experiences, but also to allow for sector-specific differences. We believe that this method was mandated as we uncovered both. The difference in how the FIM assessments were applied in the two sectors, as stated under “setting,” account for some of the sector-specific differences we found. We do not know whether these differences would have been present if the exact same setup would have been applied. The uneven professional representation among our participants, e.g., four physiotherapists and one neuropsychologist, may have influenced our findings as it is possible that we have not uncovered certain profession-specific aspects that could have been elucidated with more interviews. As a contrast, a consequence of our sampling strategy and timing of analysis is also that we may have conducted an unnecessary number of interviews for some professions as their profession-specific aspect reached data saturation with fewer interviews.

## 5. Conclusion

The use of FIM was a catalyst for improved interprofessional knowledge transfer and interprofessional collaboration within the separate teams, while intraorganisational and cross-sectoral dissemination of patient-specific knowledge was limited.

Future studies could focus on whether improved interprofessional collaboration through FIM assessments causes actual improvements in clinical practice or results in improved patient outcomes. Further, FIM assessments were highly time-consuming for the HCPs. We did not examine whether the time allocated constituted added healthcare costs or was made up for by improved and more effective communication in the interprofessional teams. These aspects were outside the scope of this study and should be explored in future studies.

## Figures and Tables

**Table 1:** Summary of data material.

	Focus group interviews		Individual interviews		Participant observations	
	Hospital	CNC	Hospital	CNC	Hospital	CNC
Number of interviews/observations	1	1	7	8	6	5
Number of participants	5	5	7	8	22	20
Duration of activity in minutes (mean)	104–119 (111)		23–60 (47)		35–57 (49)	

CNC: community neurorehabilitation centre.

**Table 2:** Example of generating themes based on a thematic analysis.

Quote	Code	Potential theme	Final theme
(OT2) “(…) Yeah, I think it would be good for, uh, interdisciplinarity, but whether there's time to assess *all* patients, I might be concerned about that. You could just make time for it, but then it's time away from something else, you see.” (PT4) “That's the key, right there (PT3: “Exactly.”; OT2: “Yeah.”). Time is what it's all about.” (I1) “Because it would be beneficial to the patient?” (PT3) “Exactly.” (OT2) “Definitely.”	Potential benefits and consequences if FIM assessments are conducted on all patients	Dichotomy of time consumption	Time

OT: occupational therapist; PT: physiotherapist; FIM: functional independence measure.

## Data Availability

The empirical material used to support the findings of this study is included within the article. The interview guides for the individual interviews as well as for the focus group interviews are available from the corresponding author upon request.
